# Workplace inequities and health outcomes among Black professionals in Canada

**DOI:** 10.1371/journal.pone.0311729

**Published:** 2025-04-08

**Authors:** Dora A. Mugambi, Karim Keshavjee, Oliver Emre Aygun, Tina K. Mbinda, Aziz Guergachi

**Affiliations:** 1 IHPME, Dalla Lana School of Public Health, University of Toronto, Toronto, Ontario, Canada; 2 Ted Rogers School of Management, Toronto Metropolitan University, Toronto, Ontario, Canada; 3 Timiskaming Area Ontario Health Team, Timiskaming, Ontario, Canada; 4 University of Manchester, Manchester, United Kingdom; 5 Department of Mathematics and Statistics, York University, Toronto, Ontario, Canada; Joaquim Nabuco Foundation: Fundacao Joaquim Nabuco, BRAZIL

## Abstract

**Background:**

Anti-Black racism in Canada remains a significant barrier to the career advancement and overall well-being of Black professionals. Despite the existence of policies and legislation aimed at reducing workplace discrimination, Black Canadians continue to face systemic racism, microaggressions, and various forms of discrimination that hinder their professional growth and contribute to a hostile work environment.

**Objective:**

This study explores the specific manifestations of anti-Black racism in Canadian workplaces, examines the physical and mental health impacts on Black professionals, and investigates the responses and coping mechanisms employed by these individuals in the face of racism.

**Methods:**

A qualitative study was conducted involving semi-structured interviews with 24 Black professionals from diverse sectors, including healthcare, information technology, academia, and public service. Participants were selected based on their professional experience and self-identification as Black. Data were collected through in-depth interviews, which were transcribed and analyzed using Leximancer^TM^ software to identify recurring themes and patterns.

**Results:**

The study identified three primary themes: (1) Mechanisms of anti-Black racism, including microaggressions, overt bias, and tokenism; (2) Impacts of anti-Black racism, such as mental health trauma, career stagnation, and exacerbation of chronic health conditions; and (3) Responses of Black professionals, including code-switching, self-preservation behaviors, and early exit from the workplace. The findings reveal that despite high academic achievement and leadership positions, Black professionals face persistent discrimination that affects their career trajectories and personal lives.

**Conclusion:**

Anti-Black racism in Canadian workplaces is deeply entrenched and continues to negatively impact the lives and careers of Black professionals. The study highlights the need for more effective diversity and inclusion initiatives that address the root causes of racism. Further research is recommended to explore the economic and psychological impacts of anti-Black racism and to develop strategies to mitigate its effects in the workplace.

## Introduction

Black professionals in Canada experience microaggressions, stigma, and discrimination in the workplace, which impact their career development, social achievement, health, and financial stability. Some researchers have argued that socially constructed definitions of race are a major factor in determining access to employment opportunities in Canada [1–3]. Other researchers have highlighted the significance of everyday racism experienced by racialized persons [4], the cumulative effect of which may serve as impediments to labor market access, job performance [5–7], job satisfaction, job retention [8], and career development [9].

In 2002, almost 25% of visible minority workers reported that they had experienced racial harassment or discrimination in the workplace [10]. The Ethnic Diversity Survey by Statistics Canada in 2003 [11] found that 56% of participants who perceived discrimination or unfair treatment found that they most encountered such treatment in the workplace, particularly during job applications and promotions. Although Black professionals are presented with “equal” opportunities to apply for the same jobs and access the same workplaces as non-Blacks, something “special for him” must be done to “equip him to compete on a just and equal basis” [12].

Black people are the third largest of the visible “minority” groups as defined under the Employment Equity Act [13]. A Boston Consulting Group (BCG) analysis of a 2019 Environics study done on behalf of the Black North Initiative showed that 83% of Black Canadians experience some form of racism, which they perceive daily [14]. Byrd refers to the concept of selective incivility, a covert form of discrimination most associated with workplace interactions [15]. Dr. Akua Benjamin first expressed the term anti‐Black racism to dialectically capture the unique nature of systemic racism that Black Canadians experience [16]. Lei and Guo state,

“Meanwhile, institutionalized structures of white supremacy in Canada that marginalize racialized communities, including Black Canadians, have never been erased. What is being erased, hidden, or silenced from the public is the recognition of Black Canadians’ experiences of marginalization and social injustice as something existing ‘here,’ ‘now,’ and ‘always’ in Canada and something rooted in and sustained by Canada’s institutional structure” [17].

A report by The Conference Board of Canada suggested that there is a tendency to preserve the status quo and the underlying racism, with employers often citing a “lack of fit” as the reason for neither hiring nor promoting skilled minority candidates [18]. Canada is not known for overt discrimination but practices a more deep-seated, nuanced, and subtle form of discrimination that leads to the same or worse impact as overt racism. For example, the perennial request for job seekers to have Canadian credentials and experience has lingered for more than 2 decades and is still ongoing. Other barriers faced by racialized individuals include discrimination for speaking with a non-white accent, having ‘foreign-sounding’ names [19], being unfamiliar with Canadian norms [20], and having foreign credentials [21]. Many workplaces also have higher performance expectations for racialized candidates [22]. The Conference Board of Canada estimated that the failure to recognize foreign credentials would cost the Canadian economy $17 billion in 2015 [23].

Despite legislation adopted to reduce discrimination in the workplace, such as The Employment Equity Act, the Canadian Human Rights Act and their respective institutions, the Canadian Human Rights Commission and the Canadian Human Rights Tribunal, visible minorities still experience racial discrimination in the workplace [24]. The heightened state of sensitivity to anti-Black racism, as first expressed in the USA [25], followed by the Black Lives Matter (BLM) movement [26], which snowballed into a global call for justice, serves to demonstrate that racial discrimination and specifically anti-Black racism persists in society and in the workplace [27,28].

Discomfort and denial of the reality of racism in the professional workplace contribute to its continued persistence and prevalence. Dr. Benjamin’s stance on anti-racism is rooted in national-scale policies from stakeholders of all sectors, including but not limited to, business corporate executives, educators, government officials, and the media [29]. Her work underscores the importance of mainstream media championing a cause to debunk the fallacy that such factors as race, ethnicity, skin color or religious background are markers of either inferiority or superiority [30–32].

## Literature review

Research on anti-Black racism in the workplace has focused on several key areas, including systemic racism, microaggressions, intersectionality, coping mechanisms, and diversity and inclusion initiatives. Systemic racism, as explored by Nkomo [33] and Bell [34], reveals how organizational policies and practices perpetuate racial inequalities, particularly in leadership representation and performance evaluations. Microaggressions [35] contribute to a hostile work environment, impacting Black professionals’ mental health and job satisfaction. Intersectionality, introduced by Crenshaw [36], highlights the compounded discrimination faced by Black professionals who also belong to other marginalized groups, such as women and LGBTQ + individuals [37,38]. Coping mechanisms, including code-switching and seeking support, are strategies employed by Black professionals, though more research is needed on organizational support for resilience [39]. Diversity and inclusion initiatives, while widespread, often fail to address the root causes of racism [40], underscoring the need for more effective approaches to creating inclusive work environments. King et al. [41] provide a comprehensive overview of the persistence and impact of anti-Black racism in organizations, offering valuable insights and considerations for future research and practice in this area.

Studies show that although workplaces recognize the importance of diversity and inclusion, forms of microaggression are rife within the workplace [42,43]. A 2019 Environics survey, reported as the first of its kind in Canada to look at race relations on a national level, found that 20% of Canadians reported experiencing racism and that 40% of their experiences with microaggression occur in the workplace [14]. The survey was conducted online between April 17 and May 6 with a sample of 3,111 Canadians 18 years of age or older. It is hypothesized that anti-Black racism is more rampant at a systemic level than is openly acknowledged in Canada. This should not come as a surprise as “polite” racism, a term coined by Martin Luther King Jr. to describe systemic racism, goes back more than 50 years through language and policies used to excuse and perpetuate racial injustice [44,45]. In addition, there is merit to Martin Luther King Jr.‘s notable shift from a focus on equality to justice, as his expressed belief was that assimilation efforts alone were not enough in order to correct the wrong dealt to and experienced by the Black community [46–48].

Several North American research studies have singled out cultural and organizational behaviors of daily discriminatory practices as one of the main reasons for high turnover amongst non-White visibly presenting workers [49,50]. This results in frustrated non-White employees leaving the workplace because of limited, reduced and/or no access to advancement opportunities. When capable non-White employees see no opportunity to advance, they tend to leave the organization [51,52]. When organizations do nothing to address this trend, it begins to affect employee morale, as the affected individuals may become less productive over time as they look to apply their knowledge elsewhere or leave the organization abruptly [53]. As a result, despite a non-White identifying professional’s high skill level, organizations lose skilled talent. Some organizations have become notorious for having the “revolving door syndrome,” unable to retain a diversity of hard-working employees [54,55].

Hiranandani states that “an antiracism approach is an action-oriented strategy for institutional and systemic change that has at its core the interrogation of privilege, power disparities, and other forms of inequity within the organization” [56]. However, this head-on approach has not had much impact. In broader efforts to diversify professional teams, muted systemic racism has continued to persist [14]. Based on lessons learned from a variety of antiracist initiatives, Hiranandani emphasizes the need for subtlety in approaching antiracism in Canada’s institutions and workplaces [56].

In spite of significant research on anti-Black racism and its impact within the workplace, there is limited documented knowledge about the experiences of Black professional workers in Canadian workplaces. Black Africans in higher academic institutions and international corporations face daunting challenges in having their knowledge recognized, getting their research funded, or having their contributions valued in the corporate world [57].

The literature on anti-Black racism in the workplace reveals several key gaps. There is a dearth of research in regions outside of North America, Europe, and South Africa, particularly in Asia and Latin America, where unique challenges may exist for Black professionals [58]. There is a need for more longitudinal studies to understand the long-term impact of anti-Black racism on career outcomes [59]. The effectiveness of equity, diversity and inclusion initiatives in reducing racism also requires further empirical evaluation [60]. The psychological and mental health impacts of anti-Black racism in the workplace remain underexplored, necessitating more focused research in this area [61]. King et al. [41] highlight the need for comprehensive research to address the persistent issues related to anti-Black racism in organizational contexts, including “specific manifestations of anti-Black racism within organizations [and] the double-bind of authenticity for Black employees.” Snyder and Mohammed [62] point out that most studies on anti-Black racism lack voice, thereby limiting our understanding of racialized experiences.

This paper fills an important gap in the anti-Black racism literature by exploring workplace racism among Black professionals working in Canada. Specifically, we study the specific manifestations of anti-Black racism [41], the health impacts, both physical and mental, of racism in the workplace [61], and the double-bind of authenticity [41]. The in-depth interviews conducted in this study ensure the authentic voices of people who have experienced anti-Black racism are captured, heard, and shared [62].

Our study explores the mechanisms of how anti-Black racism is perpetrated in the Canadian workplace, the impacts it has on Black professionals, and the range of responses and reactions that are undertaken by Black professionals in response to these insults. Digressing from the tendency of earlier research to focus on the whole spectrum of immigrants and non-White presenting minorities [63], we chose to focus on a unique cluster of Black Canadian professionals and specialists, all of whom had at least one professional designation in addition to their foundational undergraduate to doctoral university degrees. The focused cluster included but was not limited to leaders in the following sectors: Hospitals and Healthcare, Office Automation, Information Technology, Luxury Hospitality, Engineering, Academia, Process Improvement, Public Sector, and Entrepreneurship.

We set out to answer the following questions: 1) What are the specific manifestations of anti-Black racist experiences of Black professionals in the Canadian workplace? 2) What are the health impacts, both physical and mental, of anti-Black racism on Black professionals in the Canadian workplace? And 3) How do Black professionals respond to the anti-Black racism they experience in the Canadian workplace? To our knowledge, no study of this kind has been conducted in Canada. The primary purpose of this study was to, for the first time, document the recurring themes of anti-Black racism (structures, mechanisms, and impacts) experienced by Black professionals or specialists in Canada’s private, public, and non-profit employment sectors.

## Methodology

### Ethics approval and participant recruitment

The Research Ethics Board at Toronto Metropolitan University (TMU) approved the study. Participants were recruited mainly through LinkedIn, using professional connections and referrals. Invitations were sent via LinkedIn’s InMail [64] to identify recurring themes of anti-Black racism experienced by professionals in Canadian workplaces. All participants who self-identified were included in the study. We recruited 24 of the 25 participants targeted for this study.

### Inclusion/exclusion criteria

Participants were required to be at least 18 years old, working in a professional capacity for a minimum of 5 consecutive years, and self-identified as Black. Immigrants or Canadian-born individuals who did not identify as Black were excluded. Selection was independent of their workplace.

### Data collection

Semi-structured interviews were conducted with 24 key informants from August 6 to November 6, 2021, focusing on their experiences of anti-Black racism, career impact, relationships, compensation, health, social life, sense of belonging, and future outlook. Interviews were done remotely via Zoom, recorded as audio-only, and consent was verbally obtained and recorded. Interviews were conducted by DM and ranged from 1-1.5 hours in length. Consistency was ensured by having one interviewer and by ensuring that all questions on the discussion guide were covered. Participants were asked to seek counseling if the questions caused them distress. Data was collected until no new information emerged, ensuring data saturation. All data files were securely stored on encrypted, password-protected drives at TMU.

### Data analysis

Transcripts were analyzed using Leximancer^TM^ [65] to identify and situate events within the overall semantic landscape. This method highlights connections among participants’ experiences and has been used in similar studies [43,66,67] . Themes were also identified by DM and OA independently. These themes were triangulated against the themes identified by Leximancer and a final version of the themes was developed through consensus.

## Results

Despite their high academic achievement and, in many cases, work experience in senior leadership positions, almost all but two respondents admitted to experiencing discrimination because of their skin color. However, those two respondents did express that when they heard derogatory tones or language directed at other Black people, it instinctively caught their attention and bothered them. In addition, these two respondents expressed feeling ill at ease at being the “only Black man” (both are CIS male) and being subjected to many questions about how they achieved what they had, knowing that they were not particularly unique when compared to the many accomplished Black people they knew.

Black professionals, who are often considered accomplished and admired in their own Black communities or acknowledged for their professional abilities at work, still report having to contend with myriad forms of microaggressions in their places of work. Some of these include perceived derogatory comments and/or being ignored or shunned, resulting in a feeling of invisibility. The most common narrative reported was the feeling of needing to intentionally behave in a non-threatening manner and, as a result, internalize or rationalize the derived pain, which some described as “exhausting”. Others reported becoming quiet, unable to authentically express themselves but rather to ‘code switch’ (adjusting language, syntax, grammatical structure, behavior and appearance to fit into the dominant cultural context) to accommodate the threats posed by their non-Black aggressors. A few respondents with chronic conditions expressed that they noticed an exacerbation in their conditions, including bronchitis, hypertension, and migraines. However, the most common impact cited was mental health trauma, which presented in a variety of ways.

### Elucidation of lived experiences themes (experiences of anti-Black racism)

**Fig 1:**
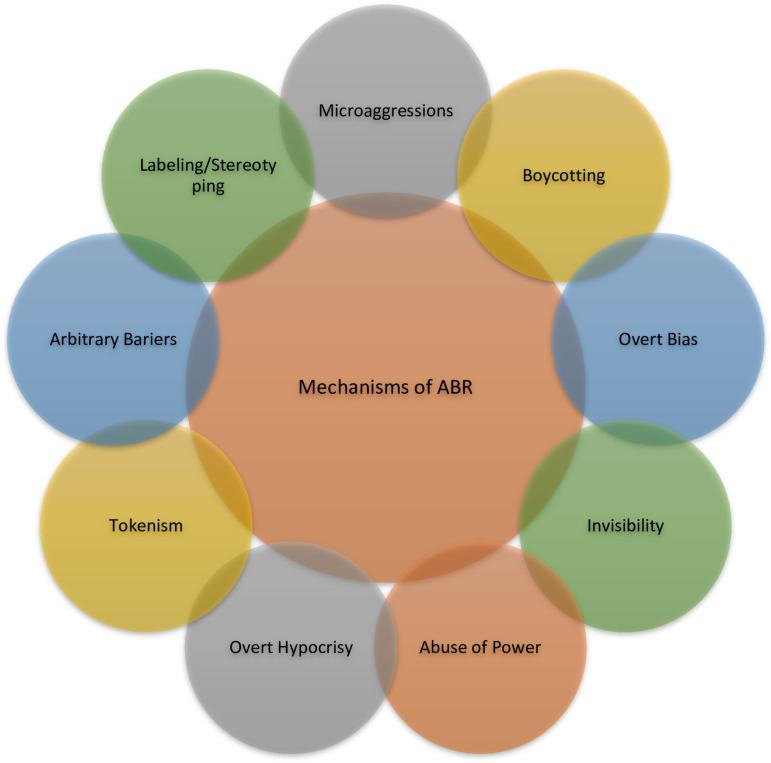
Mechanisms of anti-Black racism.

**Fig 2:**
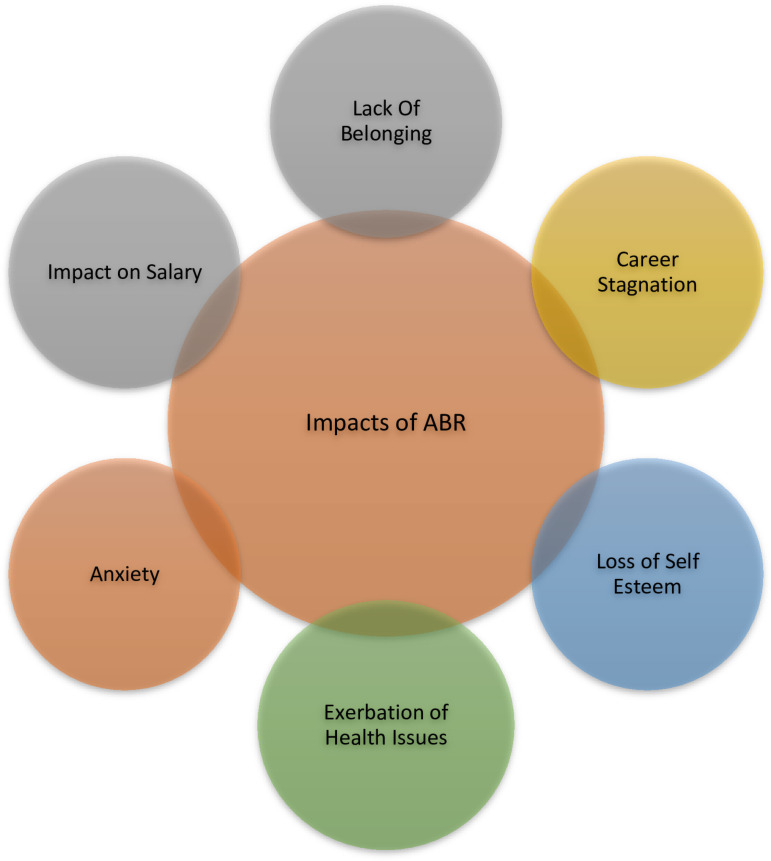
Impacts of anti-Black racism on Black professionals.

**Fig 3:**
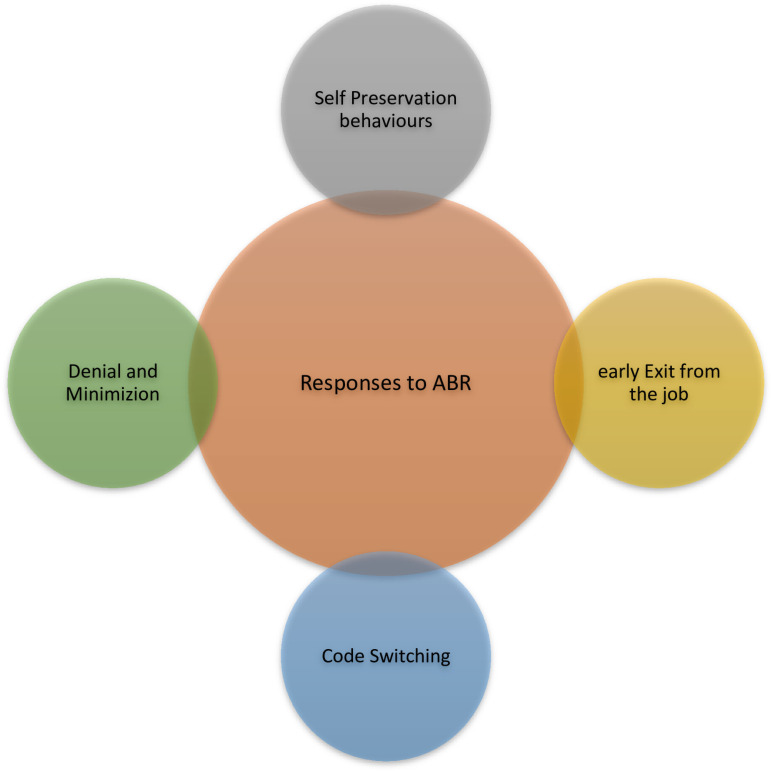
Responses of Black professionals to workplace anti-Black racism.

Analysis of the transcripts identified three major overarching themes experienced by Black professionals in the Canadian workplace: 1) Mechanisms of anti-Black racism (Fig 1), 2) Impacts of anti-Black racism (Fig 2) and 3) responses of Black professionals (Fig 3) to address the impact of anti-Black racism on their careers and personal lives.

### Mechanisms of anti-Black racism in the workplace

The following mechanisms of anti-Black racism were identified.

#### Microaggressions.

Microaggressions are the subtle, day-to-day negative attitudes and behaviors directed at marginalized groups. These can be communicated verbally, non-verbally, or through the environment. Participants openly shared their feelings about these experiences, which they encountered daily in various workplace interactions. Here are some examples of respondent quotes:

*“We feel the constant expectation for excellence, yet daily, we must keep navigating these unpleasant incidents.” –* Male engineer

*“Male staff workers kind of speak to me in a belittling and dismissive manner, during simple communication, [even when] it is not a contentious issue.”* – Male management professional

*“The microaggressions chip at me daily! I wonder if they knew just how much I was shaken by these microaggressions?”* – Female healthcare professional

#### Boycotting.

Defined as engaging in a concerted refusal to have dealings with (a person, a store, an organization, etc.), boycotting is usually used to express disapproval or to force acceptance of certain conditions.

“*My Regional Manager locked me out of his office, and he was trying to speak with my supervisor about taking my job. He was trying to fire me! “* – Male marketing and brand management strategist

*“I had 2 of my Admissions Reps who refused to do the work, so I had to speak out***.”** – Male Real Estate Entrepreneur

#### Overt bias.

Overt, or conscious, bias happens when someone is aware of their prejudices and accepts them. This type of bias is often based on a belief in a social hierarchy, which separates people into different groups and causes us to see others as inferior. Essentially, it creates an ‘us-versus-them’ mentality [68].

Some subjects experienced boldly uncomfortable situations, as depicted in the quotes below.

**“***You are speaking too loudly or just sit down – so that you are not a towering Black Body!****”* -** Male engineer.

*“The reason the patient was calling me is that she did not want me as a nurse.”* - Female Nurse

*“Especially the nurses who come from Africa get judged so harshly. It’s like George Orwell’s book - all animals are equal, but some are more equal than others* “ –Male professional working in healthcare services.

*“So, when you sit down and discuss with everybody else, and you realize, oh, they asked me to do this test. Nobody else did it. Oh, the barriers to access even casual roles because you are not one of them!”* – Male Engineer

#### Invisibility.

Invisibility is the experience of being ignored or overlooked during collaborative and social interactions, as well as being passed over for new assignments or promotions in the workplace. “...Ralph Ellison deplored the social and economic condition of this ‘invisible man’—this dark-skinned man who was not so much invisible after all, as he stood out ‘damned’ because he was Black and ‘damned’ again if he tried to integrate” [69]. Bernasconi describes how sanitation protesters in Memphis paraded with posters that read, “I AM A MAN,” asking to be recognized as human beings rather than as fixtures in the environment [70].

Our respondents echoed these sentiments with such revelations as:

*“An automatic assumption of what you are not, based on how you look; yet other people with characteristics like mine, i.e., young, and professional but white, are not automatically assumed to be junior or a student!”* ***–*** *Female healthcare strategy consultant*

“*I am in the room, and you are talking about me or around me, but not to me!”* – Accomplished female faculty and leader in organizational development

*“At events, Whites only talk to you when they need something. They will assume you are in attendant services and assume you can serve their drinks!”* – Accomplished female faculty and leader in organizational development

#### Abuse of power.

This refers to the misuse of influence, power, or authority against someone else. It is especially serious when this power is used to negatively impact someone’s career or employment conditions, such as appointments, assignments, contract renewals, performance evaluations, or promotions. Abuse of power can also involve creating a hostile or offensive work environment through intimidation, threats, blackmail, or coercion. Discrimination, harassment, and sexual harassment are particularly severe when paired with abuse of power.

Racism is a powerful tool for increasing social capital, economic power, and political influence for those who practice it [71]. It is also perpetuated in social networks by denying its existence and preventing discussions about it [72].

*“Leadership seems unaware of microaggressions, so bringing up racism is often swept under the rug and minimized”* — Female healthcare strategy consultant.

**“***My white male manager who would frequently take and present my work which he did not contribute to, was promoted. But I was NOT given a chance for promotion”* **–** Female healthcare strategy consultant.

#### Overt hypocrisy.

The Canadian workplace promises fairness and meritocracy, yet racial discrimination persists. This is confusing for those who experience it and highlights the hypocrisy in our workplace and society. Overt racism is the intentional and obvious harmful attitudes or behaviors towards a minority individual or group based on skin color [73].

*“Our attempts to form a Professional Engineering Group were resisted because our names were Black sounding*.” - Male professional engineer turned senior project manager

“*As a recruiter, I was asked to recruit people with* ***Canadian Experience, whose accents “would not get in the way.****”* - Male marketing and brand management professional

#### Lower or unequal pay/lack of promotion.

Many studies show that racially marginalized immigrants in Canada have lower earnings and higher unemployment rates than the rest of the population[74–79]. The theme of unequal pay was common among respondents, highlighting how openly discrimination in compensation can occur.

A male engineer recalled, “I was intentionally ‘low-balled’ by being placed in a lower pay bracket to reduce my internal power to bargain. I had to weigh accepting this, especially with ‘The Black Tax.’”

The Black tax refers to the obligation many Black people have to support not only their nuclear family but also friends and extended family, often due to economic imbalance. Other respondents reported this theme manifesting as an inability to be promoted. Participants lamented the number of times they experienced stagnation when staying with a single employer. Most reported no promotion opportunities in sight.

*“The root cause is stagnation (in the workplace) and finally the death of your soul.”* - Accomplished female entrepreneur with expertise in leadership coaching.

*“You choose a pattern of moving on and being accused of never staying long enough”* - Female health strategy consultant.

“*My career became a series of lateral moves, no upward mobility including pivoting into new career paths*. “- Male navy veteran, and IT consultant

#### Tokenism.

Tokenism is the practice of making a superficial effort, like hiring someone from a minority group, to avoid criticism and create the illusion of fairness [80,81].

*“Feeling like a token can be draining, especially if one also feels like “the only one”* –Seasoned male business professional and entrepreneur.

“*I landed in EDI mentorship - more of a “color celebrant” with no budget!*” *-* Female entrepreneur, former bureaucrat

“*People call me exceptional yet demographically we Blacks, and Coloreds are ‘taking over.’*- Experienced female nurse.

“*When I am with white people, I find they have a hard time considering that my skills are superior to theirs.” -* Seasoned business professional and entrepreneur

*“The DEI position had no power in the University, there was a lot of show but no operationalization of diversity in programs or other important outputs.”*- Accomplished female nurse in academic leadership.

*“I have refused a chance of promotion because the idea of being “the First one” comes with a high risk for us as Blacks. What with White females gaslighting Black men and white men sabotaging our leadership?” -* Experienced male project management professional

#### Arbitrary barriers/ higher barriers for the same job.

A variety of tactics are used to prevent Black people from getting certain jobs. These tactics are based on the personal preferences of those in charge of hiring, rather than any established industry standards. Many respondents said that constantly having to advocate for themselves and facing repeated obstacles in their professional growth, such as getting a work contract issued or renewed, can be “exhausting.”

***“****In the government, you tend to work on contracts a lot longer, and your contracts can and do run out as you wait.*” - Male engineer

*“I was told it was not possible to do my practicum while working. My white colleague was allowed to do half practicum at full pay, without any problems, so I knew that when I applied, and I said I wanted to do my practicum it would be ok, but it was not possible!”* - Female nurse and researcher

*“…. Many Black professors receive tenure and promotion to associate professor, but they rarely advance to the Full Professor ranks, despite having scholarly productivity and professional accomplishments that meet or exceed the threshold.”* - Seasoned female nurse in academic leadership

#### Labeling/stereotyping.

Humans often stereotype others, and Black people frequently face pervasive and persistent stereotypes [82]. Black men are often labeled as ‘aggressive.’ Black women face stereotypes that portray them either as happy, large, and kitchen-savvy (the ‘mammy’ stereotype) or as loud, aggressive, and opinionated (the ‘sapphire’ stereotype). In this study, we found another stereotype: geographic or neighborhood stereotyping. This involves assuming Black people come from a specific neighborhood, implying a specific (low) socio-economic class. In general, Black people are often considered ‘junior’ or less qualified based on their appearance.

### “Angry Black Woman Syndrome” (ABWS)

This widely recognized negative stereotype uniquely affects Black women. Researchers suggest that Black women are more likely to be perceived as belligerent, contentious, and as having angry personalities, a perception not as readily assigned to other men and women groups [83]. When Black women express anger at work, it often leads to their leadership being questioned. Research has found that Americans are more likely to attribute anger in Black women to personality defects rather than situational circumstances [84–86].

*“When I inquired why it was happening – I was characterized so negatively, and asked why I was questioning*. *If I don’t say it, it is just going to continue. I sometimes take truth to power and not wonder, or think “can I say this?” I just do it!” -* A female healthcare strategy consultant

### Aggressive Black man

*“In Corporate Canada, racism is a sin of omission, subtle, with microaggressions always present [...] You are speaking too loudly or sit down [...] so that you are not a towering Black Body!” -* An accomplished male engineer

### Coming from a specific geographic area or having a specific socio-economic status

*“In healthcare I found I was always the ONLY Black woman***.** *Yet I would constantly hear snide comments like I must live in “ScarBeria.”* *Using the assumption that all Blacks live in Scarborough. I was brought up in Scarborough ….”* - Female strategy consultant

### Having a lower level of competence

“*An automatic assumption of what you are or not, based on how you look; yet other people with characteristics like mine, i.e., young, and professional but white, are not automatically assumed to be junior or a student! –* Female Strategy Consultant

### Impacts on Black professionals

#### Lack of belonging/exclusion/being identified as an “other”.

*“I did not go golfing to get the insights for politics with a small “p.” As a younger worker from a different culture I was not aware of the initiative I was supposed to take to move my career forward*.” -Male research & development (R&D) chemist

*“They relate almost like a religion (the whites). I mean, right now, I’ve seen the agents rise, you know, to director positions. So, I think the Africans, we need mentorship on how to navigate our careers” – A male brand and marketing professional.*

*“Especially the nurses who come from Africa get judged so harshly. it’s like George Orwell’s book - all animals are equal, but some are more equal than others.” –Male R & D chemist*

*I felt the exclusion of being labeled not ready for promotion despite my work ethic, experience, and qualifications* —Female talent sourcing and acquisition professional.

#### Career stagnation/lack of professional advancement/frustration of ambitions.

“*The root cause is stagnation (in the workplace) and finally the death of your soul***.** *You choose a pattern of moving on and being accused of never staying long enough” —* Female entrepreneur, former bureaucrat.

*“Meritocracy – what is it? Merit of social graces and access to knowledge of that corporate culture?” — Female talent sourcing and acquisition specialist*

*“I have learned to no longer strive to climb that corporate ladder or seek external validation. I instead fulfill my purpose elsewhere, nurturing my other passions outside of work” —* Accomplished female faculty and organizational development professional

*“I changed my perspective to improve myself and not look for external validation. I have learnt how to invest in my personal development, to be excellent and yet still not getting the validation” —* Female healthcare strategy consultant.

#### Loss of self esteem.

Loss of self-esteem happens when people face repeated slights that make them feel undervalued. This emotional struggle involves inner feelings and beliefs that their talents, abilities, and character are not recognized or valued by others or by society because of racial prejudice.

The “invisibility syndrome” is a concept that helps explain the mental processes and outcomes of dealing with the stress caused by racial slights and the feeling of being invisible.

**“**T*he very first time or the second time when I did not get the job I applied for, my self-esteem just went down but with each rejection it keeps going down*. *I started wondering what is expected of me”. —* Male R & D Chemist

*“I asked my manager if I can work from home, and she told me “NO”, yet other workers received accommodations to work remotely. “—* Female Social Worker

*“Students were rude, biased, and aggressive to Black instructors, their evaluations were often unfair, written from their own points of view. I had to evaluate things overall in a very objective manner and calm down aggressive White students.” — S*easoned female nurse in academic leadership.

#### Exacerbation of health issues/worsening health.

*“I am visiting my doctor more often than in the past, (i.e., from 4 times a year to every 3 weeks). It affected my hypertension, resulting in increased medication, and more frequent blood pressure monitoring plus doing more research for different medication options” -* Male navy veteran currently an IT consultant.

*“My Bronchitis gets worse because of the constant stress.” —*Female entrepreneur, former bureaucrat

*“I experienced* pain *in my stomach, feelings of foreboding as I got ready for work.” —* Female healthcare strategy consultant

*“I had ongoing* tension *in my neck and had to see the chiropractor more frequently.” —*Seasoned male project management professional

*“I sought mental health help and was lucky to find a community of health experts of color in Canada. That really changed my life.” —*-A male navy veteran currently an IT consultant

*“I feel the* constant *headache, stress and anxiety rising when I think of work projects.” -* Female Finance Professional

*“My Digestive health deteriorated, I had to increase my medication and consult my physician more frequently for the now chronic condition. I also consult a physiotherapist to gain physical strength to cope with the endless pressure of managing students in practice and navigating an often-hostile environment.” -* Seasoned female nurse in academic leadership

#### Anxiety/easily triggered.

*“Until we get the learning, or hear otherwise, slavery continues in the bloodline and the trauma is revisited if we watch a movie, read an article, experience discrimination.” —*Seasoned female Nurse in academic leadership

*“It is when one sees it or experiences racism that it begins to cause some racism.* *I stepped in and defended it, when I should have reported it, which is what “they” would have done if the reverse was true.” —*Female biotechnology professional

*“I feel the constant headache, stress and anxiety rising when I think of work projects.”* -A female Finance Professional

#### Impact on salary.

Many Black professionals have financial responsibilities toward their extended families because their relatives often have lower earnings and need support. This situation puts Black professionals in tough negotiation positions, especially if they need a job to support several dependents. As a result, they often feel pressured to accept lower salary offers to avoid the risk of losing the job opportunity by negotiating too aggressively. This is often referred to as the “Black Tax.” Participants generally said:

1. We need to earn equitably so that we perform consistently.
2. We feel undervalued.
3. We are shafted from role to role without upward mobility.

“*I don’t feel that in the jobs there’s equity in terms of compensation for different job positions. Also, because my job was not promoted, and I had to keep reapplying for it.*” –Engineering professional

### Connectinvg impacts to mechanisms

Tying potential mechanisms to impacts can be quite challenging. Causative factors are poorly understood in this area and conducting experimental studies is clearly out of the question. This is potentially one reason why equity and diversity programs have not had a successful track record. Poor understanding of root causes leads to poor interventions. Table 1 is an attempt at linking mechanisms to impacts. This is by no means a causative link, but may have a plausible connection.

**Table 1:** Identifying the connections between impacts and their potential mechanisms.

Impact on Black Professionals	Potentially Associated Mechanisms
Lack of Belonging/Exclusion/Being Identified as an ‘Other’	Microaggressions Invisibility Stereotyping/Labeling Tokenism
Career Stagnation/Lack of Professional Advancement/Frustration of Ambitions	Lower or Unequal Pay Arbitrary/High Barriers Abuse of Power
Loss of Self-Esteem	Microaggressions Abuse of Power Lower or Unequal Pay Labeling/Stereotyping Tokenism
Exacerbation of Health Issues/Worsening Health	All mechanisms lead to chronic stress and feelings of exclusion
Anxiety and Easily Triggered	Microaggressions Overt bias Abuse of power Stereotyping/Labeling
Impact on Salary	Lack of Promotion Lower or unequal pay Overt hypocrisy/overt bias Abuse of power

### Responses

Participants reported that they responded to workplace racism in a variety of ways. Most responses were aimed at self-preservation and coping behaviors.

#### Self-preservation behaviors.

*“I’m afraid that if we talk about race then I will be labeled, and if I am labeled, I might lose my job. I am afraid because I don’t want to lose my job.”* – Female healthcare quality coach and project manager.

*“I have learnt to come into a room and speak only, when necessary, I script my responses and listen intently; as I have encountered backlash for authentic communication. I realized there is a different type of “White feminism” that attacks visible minorities. I now keep separate professional and personal social media accounts.”* – seasoned female nurse in academic leadership.

#### Early exit from the job.

*“My career became a series of lateral moves, no upward mobility including pivoting into new career paths. I landed in EDI mentorship - more of color celebrant with no budget! I eventually left the business.”* Female talent acquisition professional

*“You choose a pattern of moving on and being accused of never staying long enough”* – Female health strategy consultant.

*“I have learned to no longer strive to climb that corporate ladder or seek internal validation. I instead fulfill my purpose elsewhere, nurturing my other passions outside of work” -* Female entrepreneur, former bureaucrat.

#### Being forced to fit in (code switching).

Code Switching refers to how members of underrepresented groups adapt to fit into the dominant group [87,88]. This can involve changes in language, behavior, or dress and may be done consciously or unconsciously. For people of color, it is often seen as a matter of survival [87].

According to Cooks-Campbell, being skilled at code-switching can provide a sense of psychological safety, especially in work environments that are not naturally inclusive. Human behavior often dictates that people adapt their identity to fit into different environments. If someone’s adapted identity conflicts with their true self, they may suppress their authentic identity to fit in effectively.

Black professionals often become skilled at code-switching to cope with feeling unaccepted, undesired, and unworthy in professional environments. While it can be a necessary tactic for adapting to dominant cultures and social norms, code-switching in the context of workplace racism can harm Black identities, causing feelings of being unaccepted and unworthy of professional respect.

*“There is a diversity in the spectrum of which we code-switch. We overcompensate; we are the best dressed in the room, our dress, status, and style wearing shiny shoes, we dress up! All these little things we do… you overdress to disarm people. It is a sobering memory, you must double-think about wearing a baseball cap, especially when you are leadership, you must think twice*!” –Male business executive, morphing into an equity diversity and inclusion (EDI) champion.

*“I had low bubbling anger because of someone in my team, I became adaptive, and put my guard up but then after jumping through many hoops, I raised my voice at my team when I forgot! On the other hand, leading with empathy and sympathy is effective to a point and then it gets to where it is maladaptive, and you become like the version of yourself that is trying to make people like you*.” - Male navy veteran turned IT consultant.

#### Denial and minimization.

The difference between minimizing racism, as described by participants, and denying racism by those who don’t experience it [89] is that minimization is used as a coping mechanism and a self-preservation strategy. Minimization and denial are two sides of the same coin. Minimization, as a way to cope with racism, involves finding ways to endure in an anti-Black racist context and rendering racism and its consequences powerless. This means always being aware of one’s difference while downplaying its impact. Like non-Black denial of racism, Black minimization has several dimensions, including ignoring acts and discourses of racism, minimizing their significance, and persistently questioning whether an encounter is influenced by racialized thinking.

As researchers who share similar professional lived experiences as our participants, we recognize that Black minimization of racism is a perspective that some Black individuals adopt to deliberately disavow the social processes and institutional practices that have oppressed Black people [90,91].

Among our respondents, two distinct perspectives on racism were identified:

1. The employer knowingly ignoring what is happening internally.
2. Some Black professionals claim to be unaware of racism or to have never experienced it.

The second perspective elicited negative reactions from our research team because we also have the “unique voice of color.” Therefore, we felt it was important to objectively process, document, and try to understand all respondent perspectives, including the denial of racism.

*“In Corporate Canada, racism is a sin of omission, subtle, with microaggressions always present [...] You are speaking too loudly or sit down [...] so that you are not a towering Black Body!”* – Male Engineer

*“When your own people hang you out to dry there is a sense of betrayal. It’s concerning when Black people pretend that there is no racism when you speak of your anti-Black racism experience; they may just not yet have experienced it.”* – Male branding and marketing professional

## Discussion

Black professionals in the Canadian workplace face racial discrimination, including microaggressions, overt exclusion, bias, arbitrary barriers, and social mechanisms that do not apply to other races. They also experience lower pay for equal work and other social assaults. These feelings leave Black professionals despondent [92] and sometimes exhausted and disinterested in pursuing promotions. This chronic stress also impacts their physical health, increasing the risk of hypertension, diabetes, and obesity [93].

Microaggressions and overt anti-Black racism, as well as being othered and tokenism, are rampant in the nursing profession [94]. The impact on a Black professional’s health and wellness commonly results in emotional and mental exhaustion [94]. “Racial stratification critically impacts the quality of lifestyles and life chances of racialized groups” [95]. Kihaka states, “it is not hyperbole to suggest that America’s overt hostility toward its vulnerable citizens plays out as a ‘two-for-one deal’…where Canada’s condemnation of systemic racism ‘over there’ strategically deflects from the same ‘over here’” [96].

Our research investigates how Black professionals respond to and perceive anti-Black racism in the workplace. Three key approaches were reported:

1. To resolve their discomfort and support their families, many Black professionals engage in self-soothing activities like deflecting, code-switching, denial, and inertia. These mechanisms help them tolerate the negative attitudes and behaviors they experience.
2. When tolerance is not possible, Black professionals may leave the workplace early. Some pursue new courses or credentials to change career paths, while others opt for self-employment, accepting its risks and uncertainties.
3. Some Black professionals shift to roles in Equity, Diversity, and Inclusion (EDI), hoping for a less oppressive path. However, being the only or the first Black professional in a leadership position can be challenging due to the high visibility and associated consequences.

Historical plans to address discrimination [63] have focused on reducing discrimination or enhancing diversity by hiring “visible minorities.” While diversity initiatives aim to create a varied workforce, they often do not focus on individual parts [56]. Hiranandani suggests a more intentional approach to addressing workplace racism. Complex factors like gender and age require focused studies. We propose that anti-Black racism be considered a separate, complex factor, distinct from other forms of racism, and remain a primary focus for future research [73,74].

### Limitations of this study

This qualitative study, involving 24 key informants, provides deep insights into the lived experiences of Black professionals, the mechanisms of anti-Black racism, and the responses to it. However, the sample size is too small to generalize the findings to the entire Canadian population. Participants made recommendations on how to address their negative experiences and their impacts in the workplace, but these are not included in this report.

### Future areas of study

1. Explore the deeper connections between mechanisms and impacts. Which mechanisms impact who under what circumstances.
2. Report on recommendations to address the negative impact of anti-Black racism.
3. Conduct a quantitative study investigating the strategies and mechanisms that Black professionals use to thrive in their workplaces despite facing anti-Black racism.

## Conclusion

Anti-Black racism persists in Canada because it is treated as a relatively new concept, despite existing for generations. Dr. Frances Henry, a leading author on race and anti-racism, accuses Canada of “suffering from historical amnesia.” She states that despite evidence of pervasive and persistent racism, many White Canadians believe an “untrue but well-believed mantra” that allows them to ignore the reality of a society divided by color and ethnicity [97]. Anti-Black discrimination issues are often diluted within the broader context of ‘visible minorities,’ overshadowing the specific experiences of Black individuals.

For example, a 2016 visit from the United Nations (UN) Working Group of Experts on People of African Descent found systemic anti-Black racism in Canada’s criminal justice system, education, housing, and employment, resulting in economic disadvantages for Black people. Although a 2017 UN meeting reported satisfactory measures from Canada, the focus was on funding programs for visible minorities and indigenous people, with minimal mention of eliminating workplace discrimination [98].

This study contributes new knowledge about Black professionals in the workplace, analyzing their self-reported experiences and the impacts of anti-Black racism on their mental and physical well-being, professional journeys, and perceptions. Our findings confirm that Black individuals face significant oppression in the Canadian workplace. Most reported that their efforts and talents were actively suppressed, often leading to an early exit from the workplace. This premature departure of skilled individuals harms both companies and society at large.

The study suggests that additional research is needed to develop methods for addressing the oppression and early exit of Black professionals. Future research should focus on the economic impact of lost diversity in the Canadian workplace and identify the health and psychological consequences of anti-Black racism on Black Canadians. The urgency of this issue cannot be overstated.
